# Thymulin, free or bound to PBCA nanoparticles, protects mice against chronic septic inflammation

**DOI:** 10.1371/journal.pone.0197601

**Published:** 2018-05-24

**Authors:** Elena G. Novoselova, Sergey M. Lunin, Olga V. Glushkova, Maxim O. Khrenov, Svetlana B. Parfenyuk, Nadezhda M. Zakharova, Evgeny E. Fesenko

**Affiliations:** Institute of Cell Biophysics, Pushchino, Moscow Region, Russia; Universidade Federal do Rio de Janeiro, BRAZIL

## Abstract

In the present work, we aimed to study the effects of free and polybutylcyanoacrylate nanoparticle-bound thymulin on immune cell activity in mice with chronic inflammation. NF-κB, MAPK, and PKC-θ signaling pathway activity was assessed, alongside Hsp72, Hsp90-α, and TLR4 expression and levels of apoptosis. In addition, plasma cytokines and blood and brain melatonin and serotonin levels were measured. In mice treated with gradually raised doses of lipopolysaccharide, significant increases in the activity of the signaling pathways tested, heat-shock protein and TLR4 expression, lymphocyte apoptosis, and plasma proinflammatory cytokine levels were noted. Moreover, we observed significantly heightened serotonin concentrations in the plasma and especially the brains of mice with inflammation. In contrast, melatonin levels were reduced in the tissues examined, particularly so in the brain. Treatment of these mice with thymulin alleviated fever, reduced apoptosis, increased splenic cell number, and decreased cytokine production, Hsp72, Hsp90, and TLR4 expression, and the activity of the signaling pathways examined. In addition, thymulin partially restored brain and blood serotonin and melatonin levels. Thus, thymulin suppressed the proinflammatory response in LPS-treated mice, indicating the potential of thymulin co-therapy in the treatment of sepsis. Nanoparticle-bound thymulin was more effective in several respects.

## Introduction

Chronic inflammatory diseases and their most severe complication, sepsis, are common causes of mortality. Sepsis may occur after surgery or trauma due to massive cell and tissue death, along with immune dysfunction, and has a fatal outcome in 40–60% of cases. The most severe form of sepsis develops during infection by gram-negative bacteria, and is commonly accompanied by severe immunodeficiency. Currently available treatments for sepsis, such as antibacterial therapy using antibiotics, infusion therapy with donor fresh frozen plasma, and therapies that alleviate hypercytokinemia by decreasing levels of cytokines [particularly tumor necrosis factor (TNF)-α], are not successful in all cases. Over 40 clinical trials of treatments designed to decrease the proinflammatory response have been performed, none of which had promising results [1]. Over the last decade, preclinical and clinical studies have definitively shown that sepsis leads not only to hyperinflammation, but also impaired immunity, including dysfunction of the adaptive immune system. Certain investigators and clinical practitioners believe that the main cause of the failure of sepsis therapy involves the development of severe immunodeficiency [2, 3]; therefore, normalization of immune status may facilitate sepsis treatment.

Recent studies of sepsis, which is accompanied by multiple organ failure, have identified various stages of this severe pathology. The first stage constitutes a “cytokine storm” [4, 5], during which, an uncontrolled cytokine response leads to fever and respiratory deficiency. This condition is conventionally termed systemic inflammatory response syndrome (SIRS). The initial immune reaction is triggered by the binding of bacterial antigens to receptors of several types, resulting in the secretion of many proinflammatory molecules, such as TNF-α, interleukin (IL)-1-β, IL-2, IL-6, and interferon (IFN)-γ, as well as anti-inflammatory agents that inhibit cellular inflammatory responses. In sepsis, increased coagulation and thrombin generation and vascular hemorrhage are often observed [6]. If the patient survives the cytokine storm stage, a second, anti-inflammatory stage known as “immune paralysis” develops [7, 8]. Interestingly, a patient may encounter these stages many times during the development of sepsis [5].

It should be noted that the intensity of the initial inflammatory reaction depends on many factors, including the physical condition, health, and genetic attributes of the patient and the type of pathogen causing the infection [9]. Furthermore, uncontrolled development of the initial stage inevitably leads to immune imbalance, which increases the probability of secondary infections, such as pneumonia, and activation of latent herpes virus (including cytomegalovirus) infections. Indeed, many studies have shown that the secondary stage of sepsis is dependent on immune abnormalities, including dysfunction of the adaptive immune response [10, 11].

Apoptosis, as a programmed cell death mechanism, maintains immune homeostasis by elimination of immune cells. In this process, the caspase family of cysteine proteases are instrumental in the cleavage of cellular proteins, and the transcription factor NF-κB activates transcription of pro-apoptotic and anti-apoptotic genes. Given that NF-κB mediates hyperinflammatory responses by stimulating cytokine production, it simultaneously induces, together with caspases, apoptosis of cells of the adaptive immune system [12]. Many investigators have demonstrated increased apoptosis during the “cytokine storm” stage of sepsis both in animal models [13] and in patients with severe sepsis, who exhibit significantly decreased T, B, and dendritic cell counts [14].

There is substantial evidence that patients with SIRS and sepsis undergo severe oxidative stress, which may be a key cause of tissue damage. Moreover, levels of various antioxidants, such as glutathione, selenium, vitamin A, α-tocopherol, and ascorbic acid, have been shown to be decreased in sepsis [15]. In view of these changes, exogenous antioxidants of various types may be used to alleviate sepsis-associated oxidative damage [16]. For example, a combination of *N*-acetylcysteine and deferoxamine protects rats from the effects of sepsis [17], and we have demonstrated that a combination of liposoluble antioxidants (coenzyme Q9, α-tocopherol, and β-carotene) exerts a protective effect in a mouse model of acute inflammation induced by lipopolysaccharide (LPS) [18]. Recently, we also extensively studied the role of thymic peptides in the pathogenesis of sepsis [19]. For the first time, we revealed that the anti-inflammatory effects of such peptides are mediated by the activity of several intracellular signaling pathways [20], and that one of these peptides, thymulin, increases the anti-inflammatory activity of a specific inhibitor of the NF-κB signaling pathway, IκB kinase (IKK) inhibitor XII, in mice with acute septic inflammation [21].

Therefore, immunotherapies that stimulate the body’s defense mechanisms might comprise a reasonable approach to the treatment of sepsis. The specific biomarker-based identification of patients with impaired immunity and subsequent administration of particular immunomodulators could have great potential as a supplement to conventional sepsis therapies. Ultimately, however, the success of such treatment will depend on the defensive capabilities of the patient.

Many clinical studies of the application of immunomodulators in sepsis treatment have had disappointing outcomes, in contrast with the promising results obtained using animal models. A possible explanation for this discrepancy is that animal models are not fully representative of human pathologies, which are often complicated by many additional factors, such as age and comorbidities. Indeed, in animal models of disease, young individuals with identical genetic backgrounds tend to be used, whereas clinical trials are significantly complicated by the heterogeneity of human sepsis. Moreover, comparative gene expression studies have shown that gene transcription in mice with sepsis does not match that in humans with this condition [22].

To study the mechanisms underlying septic inflammation and the correction of immune imbalance, an adequate animal model should be used. To date, the suitability of animal models for the investigation of human diseases has been a subject of some debate. The idea that mice cannot be used to simulate severe human conditions is supported by arguments that the immune system of mice is superior to that of humans [23], and it has been suggested that divergent lifestyles have led to the evolution of differences in innate and adaptive immune responses. Indeed, animals such as mice tend to be more resistant than humans to bacterial toxins [24]. Certain authors disagree with this interpretation, however, pointing out that the mouse and human genomes demonstrate significant similarities [25]. Furthermore, the direct evaluation of novel therapies in human subjects is a clear violation of the ethical guidelines set out in the Declaration of Helsinki, which states that animal testing is required before human trials can be conducted. Therefore, the use of animal models in future studies is inevitable.

It is well known that LPS from gram-negative bacteria is a most effective sepsis inducer in animal models. To generate a chronic endotoxemia, we employed approach, consisting of daily LPS administration for 8 days, with the dose gradually being increased from 50 to 250 mg/kg of body weight. We previously applied this technique for 11 days, observing 40% mortality 1 month after ceasing toxin administration [26]. To reduce the risk of mouse mortality and “immune paralysis” in the present study, we restricted the duration of LPS treatment to 8 days. In the present work, data on the cytokine profile, fever, splenic cells apoptosis in tandem with signal and stress proteins substantially serves as markers of endotoxemia.

In addition to the immune status of mice with inflammation, we directed our attention towards the roles in this condition of serotonin and melatonin, which are secreted in the brain and other organs and are important in the pathogenesis of inflammation. Cytokines and other signaling molecules can penetrate various brain regions through the blood–brain barrier. Stress-induced hormones induce the synthesis of serotonin (5-hydroxytryptamine or 5-HT), which accumulates in thrombocytes and is released in inflammatory foci [27]. Furthermore, peripheral inflammation is known to have a direct impact on serotonin metabolism in the hypothalamus [28]. Melatonin, the precursors of which are tryptophan and serotonin, is secreted in the pineal gland, as well as many tissues and cells, including immune cells [29]. Melatonin is a natural antioxidant, a fact that has prompted many investigators to study its therapeutic potential in the treatment of proinflammatory reactions in sepsis, among other applications [30].

In the present study, we tested the effects of the thymic peptide thymulin, which has been shown to exert an anti-inflammatory influence during acute inflammation [31]. In addition, we compared the efficacy of free thymulin with that of thymulin bound to polybutylcyanoacrylate (PBCA) nanoparticles. Given that free thymulin is degraded very quickly after administration (its half-life is approximately 10 min) [9], we expected that nanoparticle-bound thymulin would be more effective in treating the chronic inflammation induced by gradually increasing doses of LPS.

## Materials and methods

### Animals, endotoxemia, and thymulin

Male 8- to 10-week-old BALB/c mice weighing 25–27 g were maintained under standard laboratory conditions (20–21°C, 10:14-h light:dark cycle, and 65% humidity) with ad libitum access to food and water. The standard food pellets used provided a balanced diet, including proteins, vitamins, and minerals. The procedures followed were approved by the ethics committee for the care and use of laboratory animals of our institution (No. 57.30.12.2011) and were in accordance with the Guidelines for Ethical Conduct in the Care and Use of Animals (Committee on Animal Research and Ethics (CARE) of American Psychological Association, APA). Mice were sacrificed using cervical dislocation and decapitated using a small animal guillotine with a sharp blade. Inflammation was induced by a daily intraperitoneal injection of LPS from *Escherichia coli* (serotype 026:B6; Sigma, USA), with the dose being increased from 0.5 to 2.5 mg/kg of body weight, in increments of 0.25 or 0.5 mg/kg, over 8 days. The total amount of LPS injected was 11.25 mg/kg of body weight. Thymulin solution (1.5 mg/kg) was injected intraperitoneally on the 1st and 5th days of LPS administration. The thymulin solution was prepared from serum thymic factor (American Peptides, USA), to which an equimolar concentration of ZnCl_2_ was added. To account for circadian changes in tissue hormone levels, mice were decapitated at 6 pm.

### PBCA nanoparticle preparation

An optimized nanoprecipitation method was used to prepare the PBCA nanoparticles [32]. Thymulin (1.0 mg) was dissolved in 10 mL 2.5% Pluronic F68 solution, and the pH of the solution adjusted to 2.5 with 0.1 mol/L HCl. Next, 50 μL BCA monomer (B. Braun, Spain) was slowly injected dropwise into the water phase while stirring. After stirring for 30 min, the pH value of the solution was adjusted to 7.8 with 0.1 mol/L NaOH, and stirring was maintained for another hour. The colloid was freeze-dried and stored at -20°C before use. The entrapment efficiency of this process has been shown to be 90% [32]. The nanoparticle-bound thymulin was administered at a dose of 1.5 mg/kg, equivalent to the quantity of pure thymulin administered.

#### Nanoparticle size and morphology

The size and morphology of the nanoparticles were examined by transmission electron microscopy (TEM) (JEM-100B; Jeol, Japan) with uranyl acetate and lead citrate contrast staining.

### Blood plasma and cells

Blood was collected during decapitation of the animals. Blood samples were kept for 3–5 h at 4°C and centrifuged at 200 ×*g*, after which, p lasma was collected for cytokine assays. Lymphocytes were isolated from the spleen in medium 199 (Sigma) containing 1% 1 M HEPES solution, 100 μg/mL streptomycin, and 10% fetal bovine serum. Erythrocytes were lysed in Tris-buffered ammonium chloride (0.01 M Tris-HCl with 0.15 M NaCl and 0.83% NH_4_Cl at a ratio of 9:1). After washing, the samples were stored at a concentration of 1×10^8^ cells/mL in RPMI 1640 medium at -20°C until needed for ELISA.

### Brain tissue samples

After decapitation, the brain was isolated, placed in a small volume of physiological solution (0.87% NaCl at pH 7.4), and kept on ice. Brain samples were then placed in phosphate-buffered saline (pH 7.4) at a concentration of 100 mg/mL and homogenized. The resulting cell suspension was subsequently subjected to two cycles of freeze-thawing, before being centrifuged at 300 ×*g* for 5 min at 4°C. The cell-free supernatants were harvested and immediately analyzed.

### ELISA

ELISA was used to determine plasma cytokine concentrations. ELISA Development Kits for mouse TNF-α, IL-6, IL-10, and IFN-γ (PeproTech, USA) were used. To visualize binding, 100 μL ABTS green dye (Sigma) dissolved in 0.05 M citrate buffer (pH 5.0) with 0.01% hydrogen peroxide was added, and optical density was measured at 405 nm using a microplate spectrophotometer (Multiskan EX; Thermo Electron Corporation, USA). To measure hormone levels, rat melatonin and rat 5-HT ELISA kits (CUSABIO, China) were used according to the manufacturer’s protocol.

### Western blot analysis

To prepare specimens, 1×10^8^ splenic cells were lysed using an ultrasonic disintegrator (UDZN-2T; Moscow, Russia) at 30–35 kHz and 50 μA for 2 min with constant stirring. The total protein concentration in each sample was then determined by the Bradford method. Subsequently, the proteins in each sample were precipitated with acetone, solubilized, boiled for 5 min, and stored at -70°C. The proteins were resolved by PAGE on a 10% gel and transferred to a nitrocellulose membrane (Amersham/GE Healthcare, UK) in a Trans-Blot chamber (Bio-Rad, USA). After blocking, the membrane was exposed to an antibody against one of the following mouse proteins for 2 h: heat shock protein (Hsp)70 [rabbit anti-Hsp72 antibody, clone SPA-812, inducible form; Enzo, USA; diluted 1:1000], Hsp90 [rabbit anti-Hsp90-α (Hsp86) antibody; Enzo; diluted 1:1000], phospho-NF-κB [rabbit anti-phospho-NF-κB p65 (Ser536) antibody; Cell Signaling Technology, USA; diluted 1:1000], NF-κB (rabbit anti-NF-κB p65 antibody; Cell Signaling Technology; diluted 1:1000), phospho-IKKα/β [rabbit anti-phospho-IKKα/β antibody (Ser176/180); Cell Signaling Technology; diluted 1:1000], IKKβ (rabbit anti-IKKβ antibody; Cell Signaling Technology; diluted 1:1000), phospho-SAPK/JNK [rabbit anti-phospho-SAPK/JNK (Thr183/Tyr185) antibody; Cell Signaling Technology; diluted 1:1000], SAPK/JNK (rabbit anti-SAPK/JNK antibody; Cell Signaling Technology; diluted 1:1000), phospho-p53 [rabbit anti-phospho-p53 (Ser46) antibody; Cell Signaling Technology; diluted 1:1000], p53 [rabbit anti-p53 (1C12) antibody; Cell Signaling Technology; diluted 1:1000], TLR4 [rabbit anti-TLR4 (M-300) antibody; sc-30002; Santa Cruz Biotechnology, USA; diluted 1:200], caspase 3 [rabbit anti-caspase-3 (8G10) antibody; Cell Signaling Technology; diluted 1:1000], phospho-protein kinase C (PKC)-θ [anti-phospho-PKC-θ (Thr538) antibody; Cell Signaling Technology; diluted 1:1000], PKC-θ (rabbit anti-PKC-θ antibody; Cell Signaling Technology; diluted 1:1000), phospho-p38 [rabbit anti-phospho-p38 mitogen-activated protein kinase (MAPK) (Thr180/Tyr182) (D3F9) XP antibody; Cell Signaling Technology; diluted 1:1000], or p38 (rabbit anti-p38 MAPK antibody; Cell Signaling Technology; diluted 1:1000). After washing, the membrane was incubated for 1 h with a biotinylated goat anti-rabbit antibody [Biotin-SP (long spacer) AffiniPure Goat Anti-Rabbit IgG (H+L); Jackson ImmunoResearch, USA; diluted 1:200,000], before being exposed to 0.1 μg/mL peroxidase-conjugated streptavidin (Jackson ImmunoResearch) for 1 h. To control for variations in protein loading, an antibody raised against a synthetic peptide corresponding to an amino acid sequence near the carboxy-terminus of human glyceraldehyde-3-phosphate dehydrogenase (GAPDH) was used [rabbit anti-GAPDH (14C10) monoclonal antibody; Cell Signaling Technology; diluted 1:1000]. ECL Plus chemiluminescence reagents (Amersham/GE Healthcare) and Hyperfilm ECL (Amersham/GE Healthcare) were then used to develop the blots according to the manufacturer’s instructions. The developed films were observed with a Vilber Lourmat (France) TFX-35.WL transilluminator. Quantitative evaluation of protein bands was subsequently performed using the computer program QAPA Ver. 3.7 (Russia).

### Statistical analysis

Statistical analysis was performed using Statistica 6.0 software (StatSoft, USA). One-way analysis of variance, followed by a post-hoc Fishers Least Significant Difference (LSD) test, was used to determine the significance of differences among groups, with p-values≤0.05 being considered significant. All values are expressed as means (± SE).

## Results

### Nanoparticles

**Fig 1:**
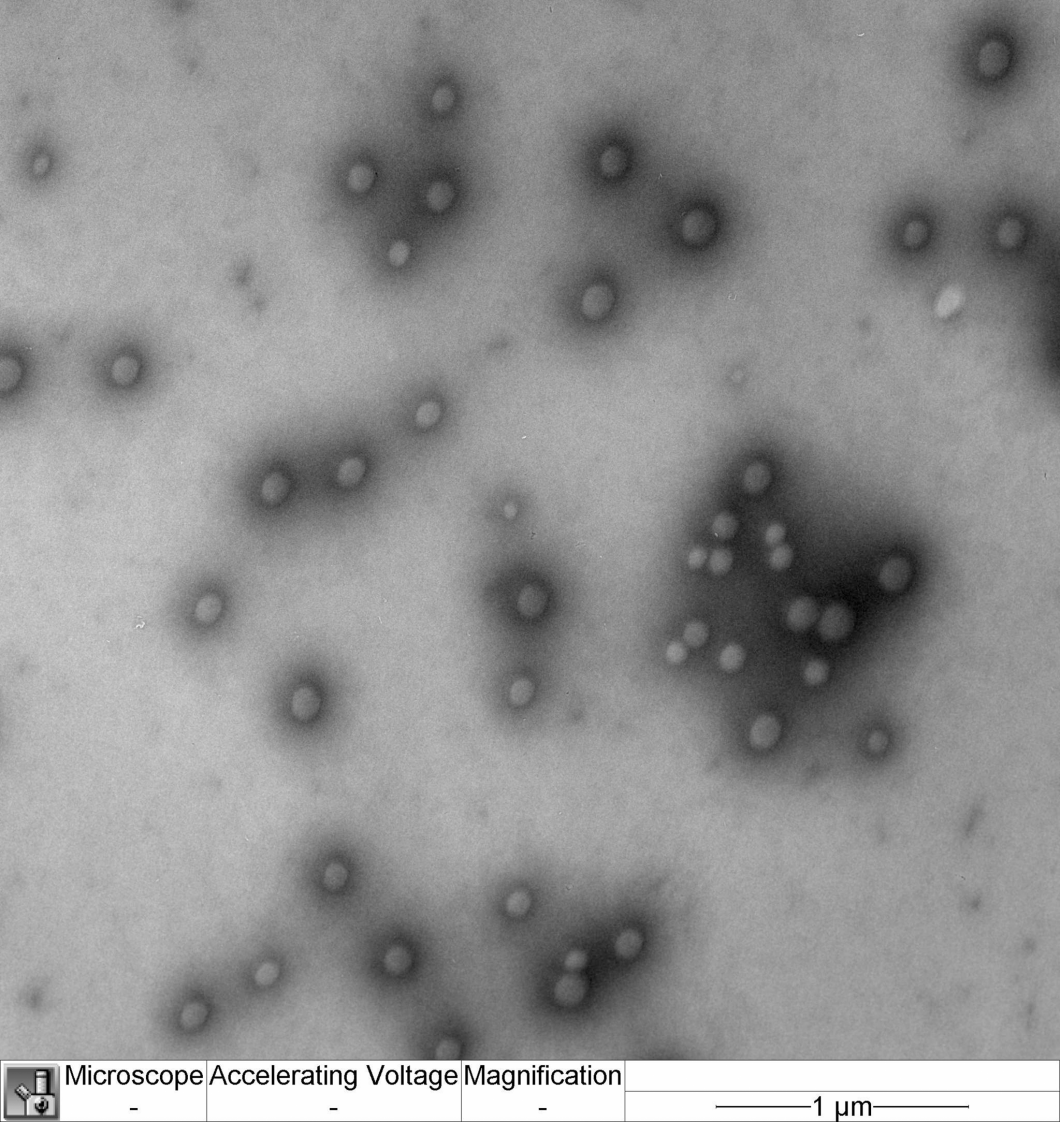
TEM micrograph of the PBCA nanoparticles obtained. The scale bar represents 1 μm.

The size and morphology of the nanoparticles are shown in Fig 1.

### Body temperature and splenic cell count

Rectal temperature was measured throughout the study. As expected, chronic inflammation led to an increase in body temperature; however, administration of thymulin, especially that bound to nanoparticles, was found to alleviate fever. Temperature measurements taken on the 8th day of chronic inflammation, immediately prior to decapitation, revealed a significant increase in body temperature at this time point (Table 1). Administration of nanoparticle-bound thymulin decreased body temperature to a level similar to that observed in the control group.

**Table 1:** Splenic cell number and body temperature of mice on the 8th day of LPS injection.

Group	Cell number, 10^6^/mg of spleen	Body temperature, °C
C	1232±44.2	37.2±0.37
IB	923±47.2*	39.2±0.4*
IB+TM	1104±49.4*	38.4±0.28*
IB+TMN	1348±58.2^#	38.0±0.4^
PBCA	1294±12	37±0.4

Controls, C; inflammation-bearing mice, IB; IB+thymulin, IB+TM, IB+ nanoparticles-bound thymulin, IB+TMN; nanoparticles, PBCA. N = 5 for each group.

*Significant difference compared to the control group, p<0.05.

^Significant difference compared to the IB group, p<0.05.

#Significant difference compared to the IB+TM group, p<0.05.

According to our data, chronic inflammation did not affect body weight, but did influence spleen weight and splenic cell number. Splenic cell counts, measured per unit of spleen weight (Table 1), were significantly lower in the chronic inflammation model. Nanoparticle-bound thymulin not only restored the number of splenic cells, but also increased it to above control values. Since nanoparticles lacking thymulin did not cause a significant change in the splenic cell count (Table 1), they are unlikely to be immunogenic.

Thus, our evaluation of physiological markers of the general condition of mice with chronic inflammation showed that thymulin, free or bound to nanoparticles, exerted a marked normalizing effect, reducing fever and preventing total splenic cell loss.

### Blood cytokines

**Fig 2:**
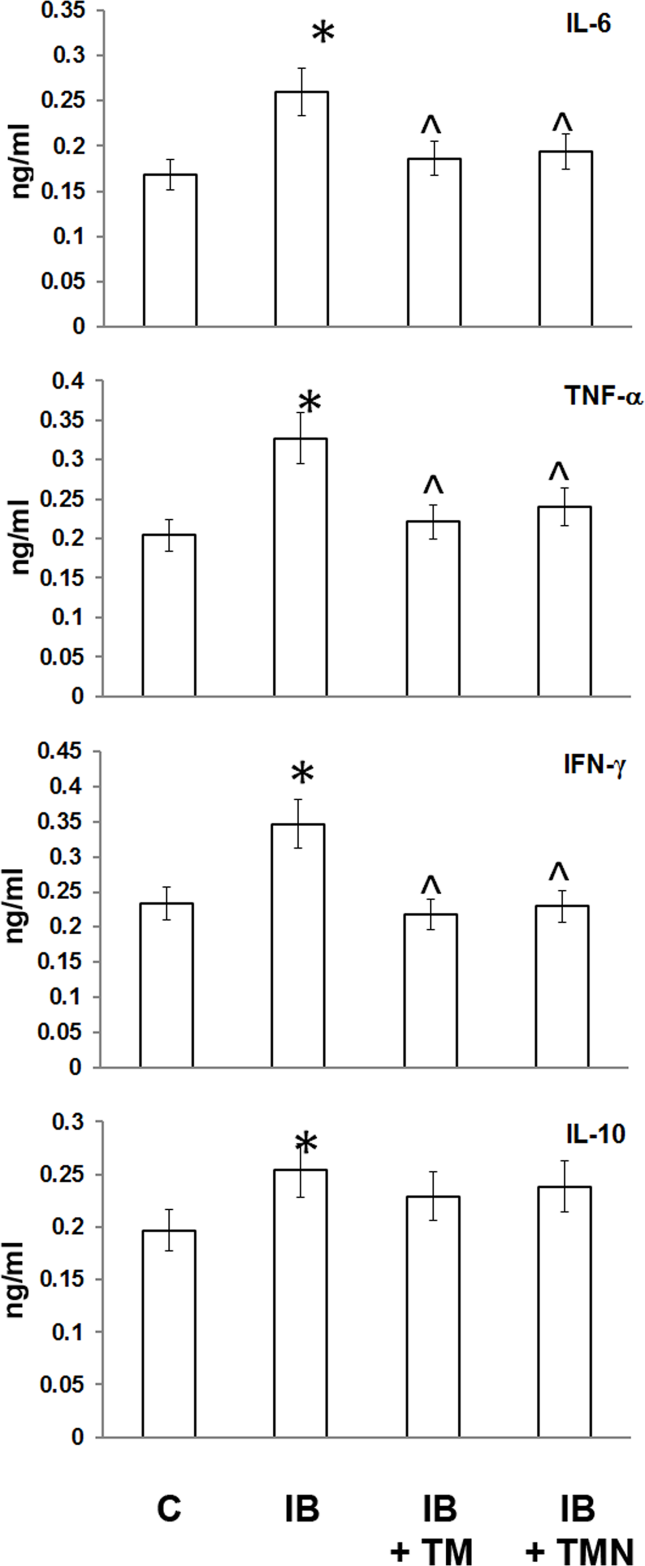
Plasma cytokine levels in mice with inflammation treated with free or PBCA nanoparticle-bound thymulin. Four groups were included: untreated inflammation-bearing (IB) mice, IB mice treated twice with 1.5 mg/kg thymulin (IB+TM), IB mice treated twice with the same concentration of thymulin bound to nanoparticles (IB+TMN), and sham-treated controls (C). There were five mice in each group, and nine replicates per mouse. The mean of the average of these replicates is shown. All animals were analyzed individually and simultaneously. The units are ng/mL of plasma. *Significantly different from the control group (p<0.05). ^Significantly different from the IB group (p<0.05).

Significant increases were noted in blood levels of proinflammatory cytokines, including TNF-α, IL-6, and IFN-γ, and the anti-inflammatory cytokine IL-10 in mice with inflammation (Fig 2). These increases were moderate, representing approximately 1.4- to 1.7-fold changes compared to the control values, indicating that at the time point studied, endotoxemia had not reached the “cytokine storm” stage characterized by much larger peaks in cytokine production. On the other hand, cytokine overproduction would also be absent during the immune paralysis stage, as cells have lost this ability by this point.

We observed that the effectiveness of free thymulin in lowering plasma proinflammatory cytokine levels was approximately equivalent to that of nanoparticle-bound thymulin.

### Signaling pathway activity

**Fig 3:**
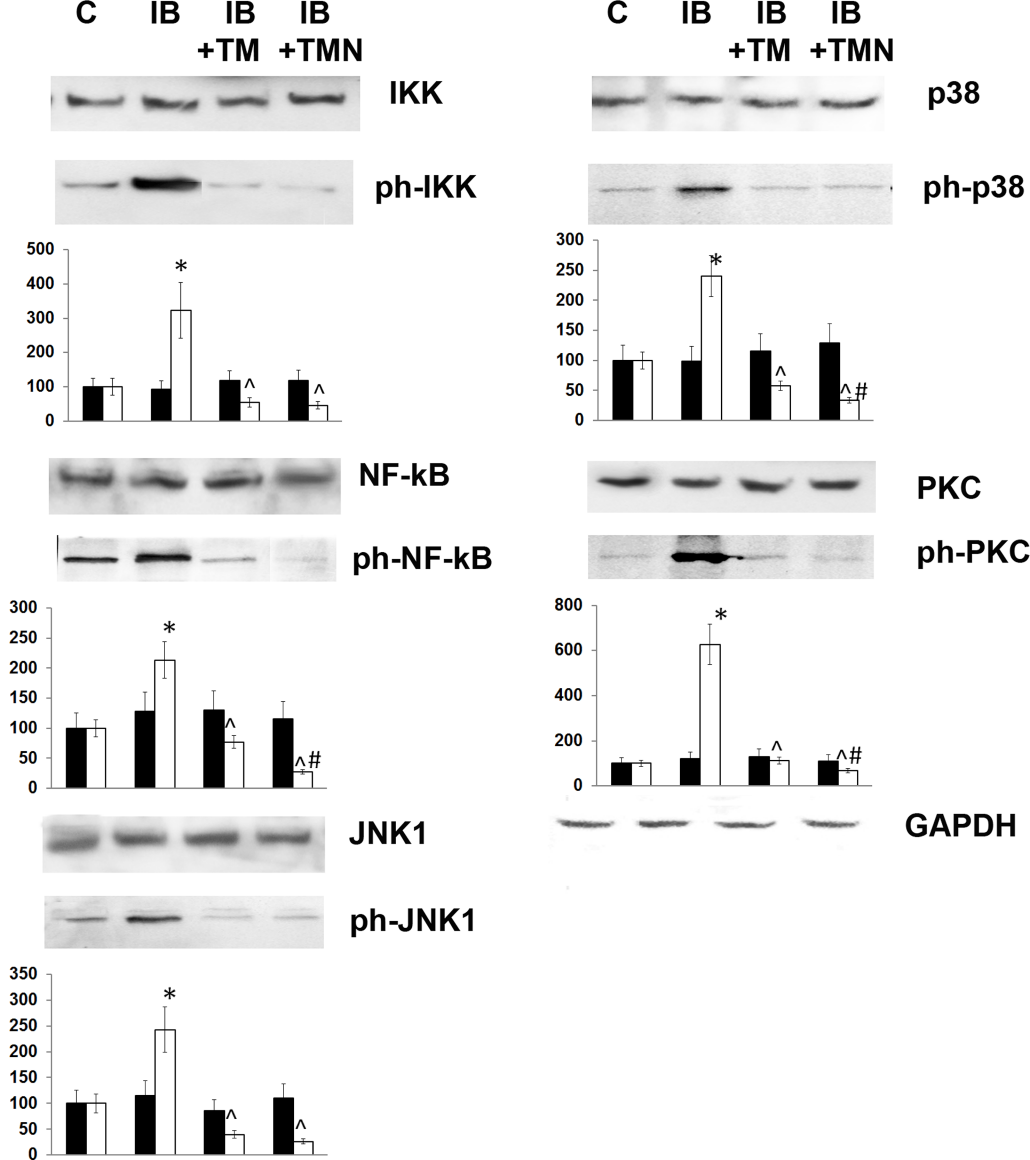
Effects of thymulin (free or bound to PBCA nanoparticles) on levels of phosphorylated and non-phosphorylated p65, IKK, JNK1, p38, and PKC-θ in splenic lymphocytes from mice with inflammation. The groups used in this experiment were the same as those in Fig 2 (1, control; 2, IB mice; 3, IB+TM; 4, IB+TMN). Western blot analysis of protein extracts from isolated mouse lymphocytes was performed using the indicated antibodies or an anti-GAPDH antibody (bottom). Images of blots show a single representative experiment using one animal. The histograms below the protein bands show quantified protein levels expressed in mean relative units (relative to the internal control GAPDH), and are the results of blot densitometry performed using the program QAPA and data from five mice. Black columns represent non-phosphorylated forms, and clear columns phosphorylated forms. *Significantly different from the control group (p<0.05). ^Significantly different from the I group (p<0.05). #Significantly different from the IB+TM group (p<0.05).

Along with elevated cytokine levels, we observed that the transitional stage of endotoxemia was characterized by activation of critical signaling cascades, as indicated by the phosphorylation of signaling proteins (Fig 3).

In particular, NF-κB signaling was found to be activated during this stage of chronic inflammation. To evaluate the status of such signaling, we tested the canonical and noncanonical NF-κB pathways via phosphorylation of IKK and p65 (a subunit of NF-κB), respectively, finding that both were activated by chronic inflammation. The JNK and p38 MAPK signaling pathways were also markedly activated in mice on the 8th day of inflammation. Furthermore, levels of phosphorylated PKC-θ, which is involved in T cell receptor signaling and NF-κB activation, were notably elevated in mice with chronic inflammation, being 6-fold higher than those in the control group. This increase was much greater than that observed in MAPK and NF-κB activation (Fig 3). Notably, administration of thymulin, especially that bound to nanoparticles, prevented the chronic inflammation-induced activation of these signaling pathways.

### Expression of TLR4, Hsp72, and Hsp90-α

**Fig 4:**
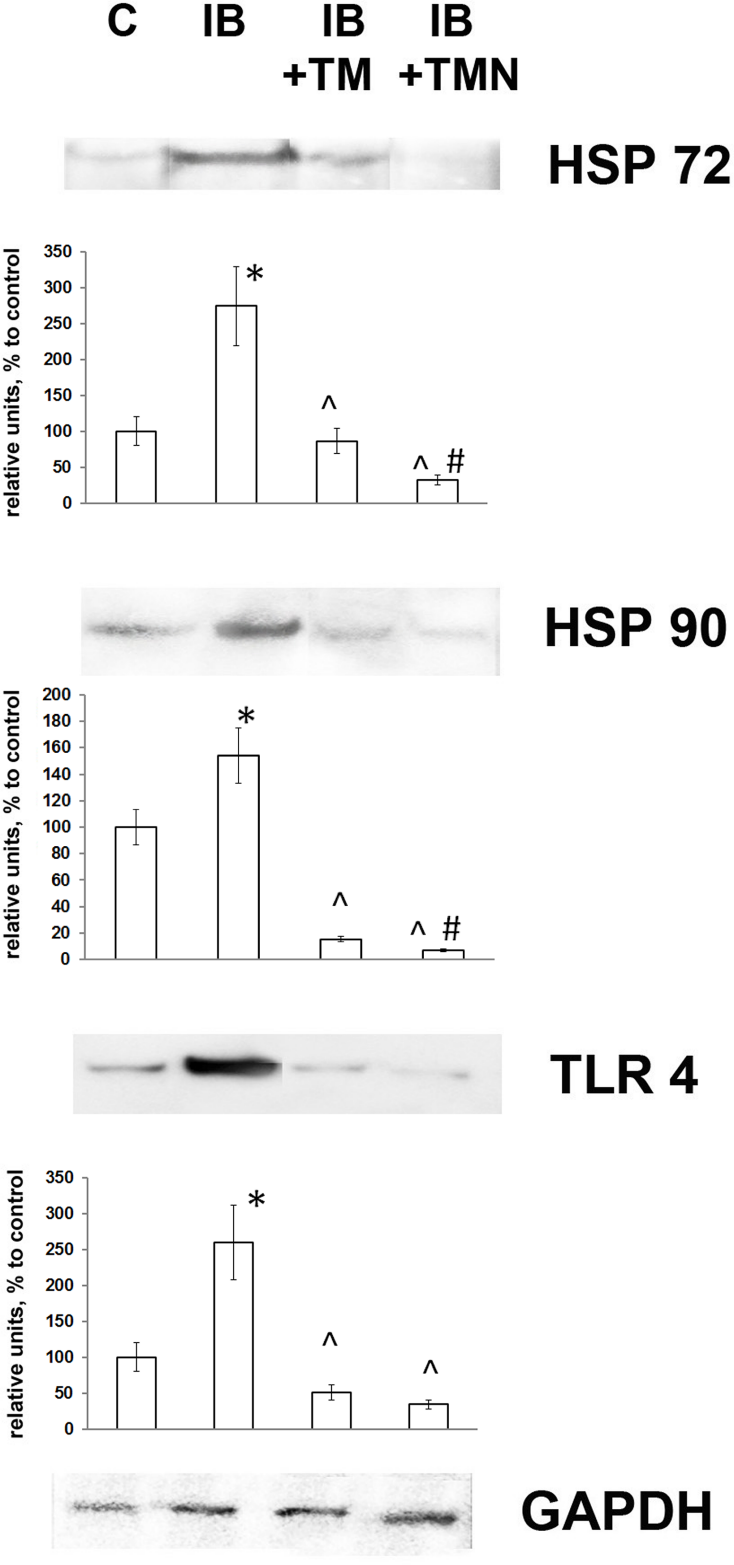
Effects of thymulin (free or bound to PBCA nanoparticles) on TLR4 and Hsp expression in splenic lymphocytes from mice with inflammation. The groups used in this experiment were the same as those in Figs 2 and 3 (1, control; 2, IB; 3, IB+TM; 4, IB+TMN). Western blot analysis of protein extracts from isolated mouse lymphocytes was performed using the indicated antibodies or an anti-GAPDH antibody (bottom). Images of blots show a single representative experiment using one animal. The histograms below the protein bands show quantified protein levels expressed in mean relative units (relative to the internal control GAPDH), and are the results of blot densitometry performed using the program QAPA and data from five mice. *Significantly different from the control group (p<0.05). ^Significantly different from the IB group (p<0.05). #Significantly different from the IB+TM group (p<0.05).

On the 8th day of chronic inflammation, a clear increase in the expression of the inducible forms of Hsp72 and Hsp90 was observed, consistent with our previous results [26]. However, in the present study, for the first time, we observed that thymulin inhibited the synthesis of both of these Hsps and that this effect was more pronounced when it was bound to PBCA nanoparticles (Fig 4). It should be noted that expression of the receptor TLR4, which specifically binds LPS and is an important marker of the inflammatory response, was also increased during the stage of endotoxemia studied, indicating hyperinflammation. However, administration of thymulin prevented this increase in TLR4 expression, with the nanoparticle form being particularly effective in this respect.

### Apoptosis of splenic cells

**Fig 5:**
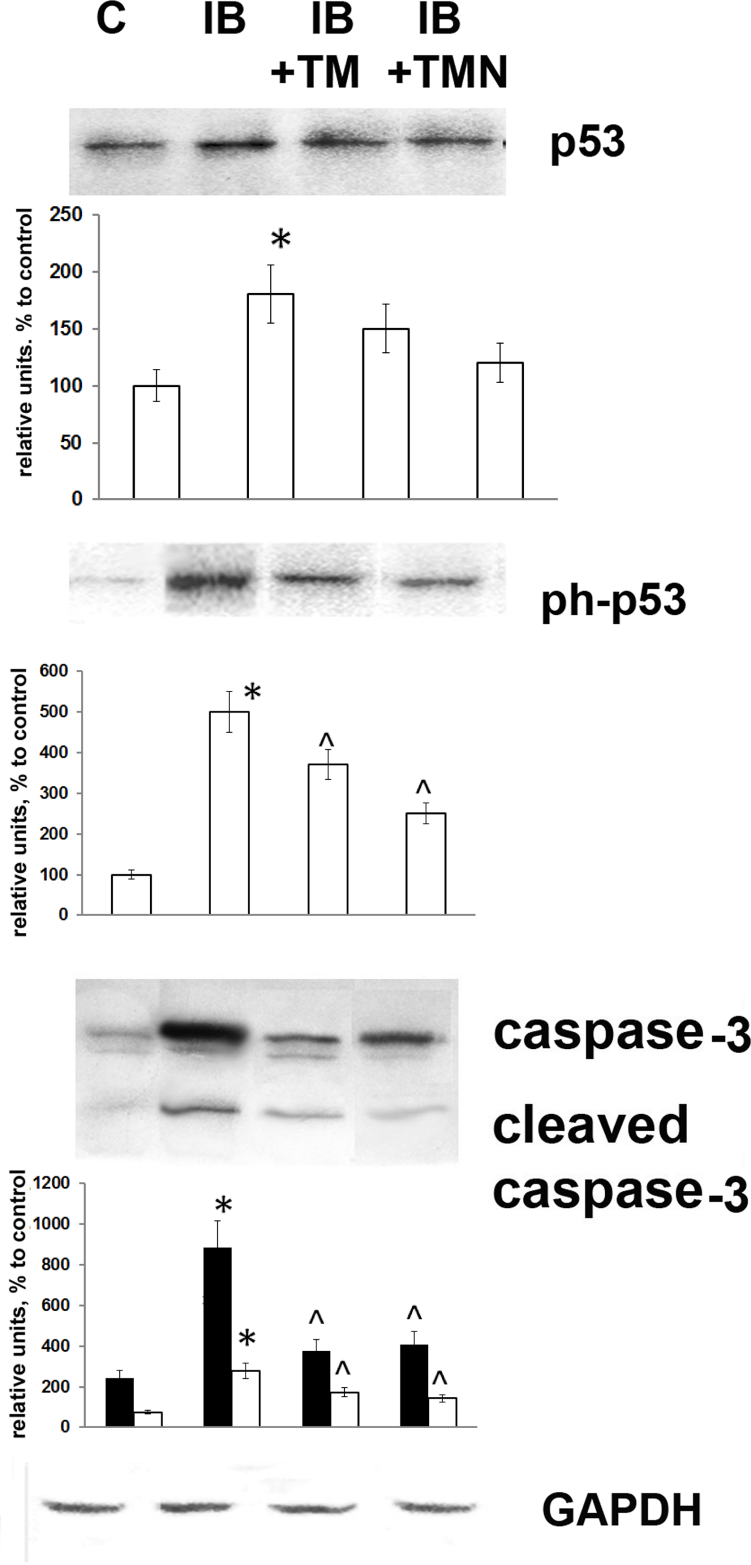
Effects of thymulin (free or bound to PBCA nanoparticles) on apoptosis of splenic lymphocytes from mice with inflammation. The groups used in this experiment were the same as those in Figs 2 and 3 (1, control; 2, IB; 3, IB+TM; 4, IB+TMN). Western blot analysis of extracts from isolated mouse lymphocytes was performed using the indicated antibodies or an anti-GAPDH antibody (bottom). Images of blots show a single representative experiment using one mouse. The histograms below the protein bands show quantified protein levels expressed in mean relative units (relative to the internal control), and are the results of blot densitometry using the program QAPA and data from five animals. Black columns represent caspase-3 and clear columns cleaved caspase-3. *Significantly different from the control group (p<0.05). ^Significantly different from the IB group (p<0.05).

Apoptosis is a critical process of controlled cell death intrinsically linked to organismal survival. Central to this process are apoptotic caspases, enzymes involved in a signaling cascade that converts danger signals, conveyed via initiator caspases, into activation of the executioner caspase caspase-3. In the present study, we measured caspase-3 levels as a marker of apoptosis. The level of this enzyme was significantly heightened in splenocytes by the 8th day of inflammation, being three times greater than that in control mice (Fig 5). In addition, a similar increase in cleaved caspase-3, considered a reliable marker of cells undergoing or having undergone apoptosis [33], was noted. Thymulin prevented these increases in caspase-3 and cleaved caspase-3 levels, independently of the form in which it was administered (free or nanoparticle-bound). We also measured levels of phosphorylated and inactive p53 (Fig 5). The observed increase in both caspase-3 and the ratio of phospho-p53 to total p53 demonstrated that endotoxemia induced apoptosis in splenic lymphocytes; however, free or PBCA nanoparticle-bound thymulin efficiently reduced the apoptosis rate.

### Blood and brain serotonin and melatonin levels

**Fig 6:**
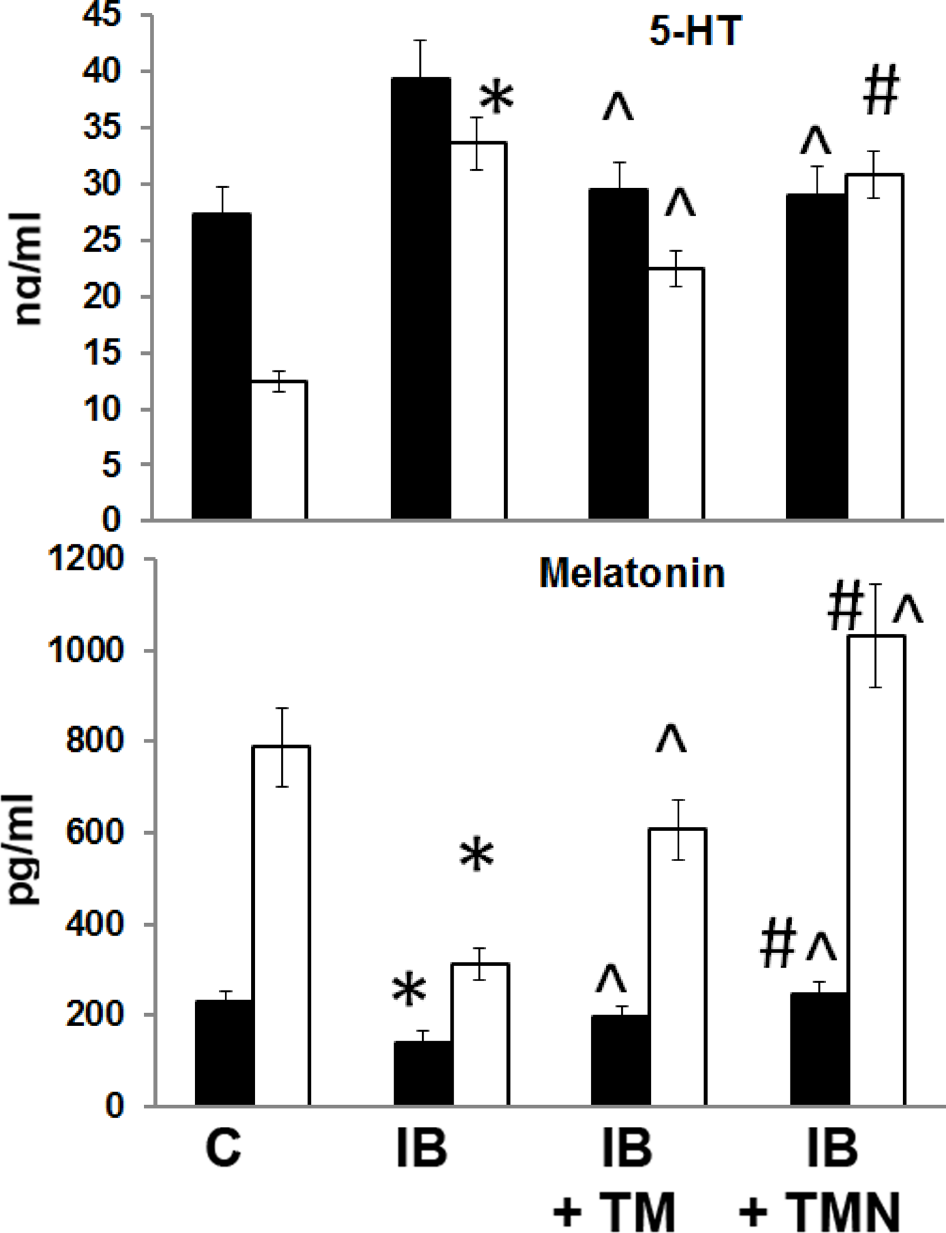
Effects of thymulin (free or bound to PBCA nanoparticles) on plasma and brain melatonin and serotonin levels in mice with inflammation. The design of this experiment was the same as that described in Fig 2. Melatonin and serotonin values are expressed in pg/mL and ng/mL, respectively. Black and clear columns represent plasma and brain concentrations, respectively. *Significantly different from the control group (p<0.05). ^Significantly different from the IB group (p<0.05). #Significantly different from the IB+TM group (p<0.05).

Plasma and brain serotonin and melatonin concentrations in mice of the four experimental groups were measured. In the tissues of mice with chronic inflammation, elevated levels of serotonin were observed, and this effect was most pronounced in brain tissue (Fig 6). Thymulin, free or bound to nanoparticles, completely normalized plasma serotonin concentration. Moreover, free thymulin significantly decreased, but did not entirely restore, serotonin levels in the brains of mice with inflammation.

Reduced melatonin levels were evident in both the blood and brain tissue of mice with inflammation (Fig 6). Thymulin administration normalized blood and brain melatonin concentrations, and the nanoparticle form was more effective in this regard.

## Discussion

Sepsis is characterized by an excessive host response to infection that is often associated with multiple organ failure and death, and there is much evidence of a major role for dysregulation of the immuno-inflammatory response in sepsis and SIRS. Such dysregulated immune reactions are associated with hyperinflammation and long-term immune suppression. Both pre-clinical and clinical studies over the past decade have demonstrated that sepsis not only involves hyperinflammation, but also simultaneously leads to impaired immunity, including adaptive immune system dysfunction [34]. Despite advances in our understanding of the pathophysiology of septic shock, its treatment remains largely symptomatic and supportive. To date, antibiotics and fluid resuscitation remain the core supportive therapies, and there is a vital need to identify and develop novel, targeted sepsis treatments. Indeed, despite recent progress in critical care therapy, sepsis is still associated with a high mortality rate (approximately 40–50%) [35]. Under normal conditions, an equilibrium exists between pro- and anti-inflammatory mediators, but this balance is disrupted in sepsis, resulting in profound changes in the relative levels of such factors [36]. The majority of researchers agree that, when combined with conventional practice, administration of immunotherapy during the appropriate immune phase of sepsis potentially represents a major advance in the treatment of this condition [1, 2].

LPS, a gram-negative bacterial cell wall component, is the agent predominantly responsible for triggering sepsis. Although multiple mammalian receptors for LPS have been identified, the most important of these appears to be TLR4, which plays a key role in the immune response to infections with gram-negative bacteria [37]. In the present work, we aimed to study the effects of thymulin on immune cell activity in a mouse model of chronic inflammation induced by daily treatment with increasing doses of LPS. Based on our earlier investigations, in which thymulin was found to have anti-inflammatory properties in vitro [21] and in acute inflammation in vivo [31], we concluded that it might exert a protective effect against the consequences of sepsis. It is important to emphasize that when applied to healthy mice, thymulin does not affect plasma levels of proinflammatory cytokines, synthesis of stress proteins (Hsp70 and Hsp90), or cellular production of nitric oxide [31]. Thus, thymulin has no effect on the inflammatory status of healthy animals, but does correct immune imbalances in chronic inflammation.

In the current work, we analyzed cell signaling, cytokine expression profiles, Hsp production, and apoptosis, with a focus on the spleen, brain, and blood plasma. Activity of the NF-κB, MAPK, and PKC-θ signaling pathways was tested in splenic cells, as was Hsp72, Hsp90-α, and TLR4 expression and apoptosis. In addition, plasma cytokine concentrations were evaluated, and melatonin and serotonin levels in blood and brain tissue were measured.

The evolutionarily conserved NF-κB intracellular signaling pathway acts as a critical immune sensor, and the mechanisms of its activation have been extensively studied. The transcription factor NF-κB is a central component of the cellular response to damage, stress, and inflammation [38]. Cytoplasmic NF-κB exists as a dimer, most commonly as the p65/p50 heterodimer, which is maintained in an inactive state by sequestration by IκB proteins. Canonical NF-κB activation is mediated by the upstream protein IKK, a heterotrimer consisting of the IKKα and IKKβ catalytic subunits and the IKKγ regulatory subunit [39]. In response to a variety of factors, including oxidative stress, IKK is activated and phosphorylates IκB, which is then polyubiquitinated and subsequently degraded by the proteasome. The degradation of IκB allows NF-κB to translocate to the nucleus, where it binds its cognate DNA sequence to regulate gene expression. Noncanonical NF-κB activation involving phosphorylation of Rel proteins, of which dimeric NF-κB is composed, was also studied in the present work.

Here, we demonstrated significant activation of the NF-κB cascade in splenic cells on the 8th day of LPS treatment, via phosphorylation of IKKα/β and p65 proteins. These results indicate activation of both the canonical and non-canonical NF-κB pathways. This is in keeping with the general view that NF-κB activation plays a critical role in LPS-induced immune responses. Thymulin, especially in nanoparticle form, prevented NF-κB activation by decreasing the activity of both canonical and noncanonical pathways.

MAPKs are a family of ubiquitous, proline-directed, protein serine/threonine kinases that participate in signal transduction pathways controlling intracellular events. We have shown here that the p38 MAPK cascade and JNK MAPK pathway were activated in LPS-treated mice. It has previously been suggested that the mechanism underlying p38 phosphorylation is distinct from that responsible for phosphorylation of ERKs and JNKs [40]. However, our results show that chronic inflammation induced similar levels of p38 MAPK and JNK MAPK activation, and that this effect was successfully prevented by thymulin application, almost independently of the form in which it was administered. Therefore, both pathways appear to constitute therapeutic targets in systemic chronic inflammation. It has been demonstrated that MAPKs and NF-κB can act synergistically to induce proinflammatory cytokine expression and release [41, 42]. Thus, treatment with thymulin to inhibit NF-κB and MAPKs may have a therapeutic effect in inflammatory diseases.

Of the signaling pathway components studied in the current work, PKC-θ exhibited the strongest activation in LPS-treated mice. Moreover, nanoparticle-bound thymulin was more effective than free thymulin in decreasing PKC-θ phosphorylation (Fig 3). These data are consistent with the role that PKC-θ is known to play in macrophage-mediated immune responses during bacterial infections [43]. Administration of thymulin, especially in nanoparticle form, prevented excessive PKC-θ activation.

TLR4 was the first mammalian TLR to be identified and was initially characterized as a pattern recognition receptor in experiments using LPS-resistant mice [44]. We noted a significant increase in TLR4 expression in splenic cells from LPS-treated mice in the present study. Furthermore, as shown in Fig 4, inflammation induced Hsp72 and Hsp90-α expression in splenic lymphocytes by the 8th day of LPS administration. These significant increases in TLR4 and Hsp expression indicate that over the period studied, the “immune paralysis” phase of sepsis was not reached. Importantly, thymulin had a notable rescue effect on TLR4 and stress protein levels, particularly when administered in nanoparticle form.

Apoptosis has been established as a critical response to bacterial infection, ultimately resulting in the removal of compromised cells. This process is orchestrated by caspases, a family of cysteine proteases that cleave their substrates at bonds on the carboxy-terminal side of specific aspartic acid residues. These proteases are generally inactive in healthy cells, being present as zymogens, but undergo autolytic cleavage when stimulated to become fully active [33]. Apoptosis allows the elimination of perceived threats to survival by directly affecting cells representing an immediate risk. Apoptotic caspases are key to this process, forming a signaling cascade that converts danger signals conveyed by initiator caspases into activation of caspase-3, an executioner caspase [45]. Cleaved caspases are responsible for most apoptosis-associated proteolysis, and the detection of cleaved caspase-3 is considered a reliable marker of cells that are dying or have died due to apoptosis. To detect apoptosis among splenic lymphocytes, we measured levels of cleaved and non-cleaved caspase-3. The observed increase in both caspase-3 forms demonstrated that the rate of apoptosis was heightened in cells from mice in which inflammation had been induced. Interestingly, the increase in non-cleaved caspase-3 was more pronounced than that in the cleaved form. Both forms of thymulin decreased splenic lymphocyte apoptosis.

Along with caspase-3, we also evaluated levels of p53, which is known to have an important function in the cellular response to DNA damage, genomic aberrations, and other apoptotic features. DNA damage induces phosphorylation of the serine residues at positions 15 and 20 within p53 and leads to reduced interaction between this protein and its negative regulator, the oncoprotein MDM2 [46]. Phosphorylation of p53 also affects its ability to induce apoptosis. The observed increases in caspase-3 and the ratio of phospho-p53 to total p53 indicated heightened levels of apoptosis among the cells of mice with inflammation (Fig 5). However, administration of thymulin, whether in a soluble form or bound to nanoparticles, decreased such LPS-induced apoptosis. These findings correlated with the number of splenic cells, which was also decreased in LPS-treated mice and restored by thymulin treatment (Table 1).

As proinflammatory cytokine expression is known to be regulated by the NF-κB and MAPK pathways, the increase in plasma levels of such cytokines (e.g., IL-6, IFN-γ, and TNF-α) after LPS treatment in the present study was expected. Plasma levels of the anti-inflammatory cytokine IL-10 were also elevated, but to a lesser extent (Fig 2). This finding is in keeping with our previous report, which demonstrated an increase in the production of various cytokines, including IL-10, in mouse cells in vivo after administration of LPS [26]. Thymulin treatment completely normalized proinflammatory cytokine levels, independently of the form used (free or nanoparticle-bound).

Accumulating evidence indicates close, bidirectional communication and a mutual regulatory relationship between the neuroendocrine and immune systems. Interactions between the endocrine and immune systems have been shown to contribute to the pathophysiology of certain conditions, including sepsis [47]. Therefore, we tested levels of the hormones melatonin and serotonin, which share tryptophan as a precursor, in the blood plasma and brains of mice with inflammation. Melatonin is a highly conserved molecule found in organisms ranging from unicellular life forms to humans. In vertebrates, melatonin is the main secretion of the pineal gland, from which it is released into the blood [48]. Endogenous melatonin, a chronobiotic regulator of endocrine and non-endocrine rhythms, is synthesized from tryptophan via serotonin by this gland. Other functions of melatonin, including its antioxidant and anti-inflammatory properties, genomic effects, and modulation of mitochondrial homeostasis, are associated with the redox status of cells and tissues [30, 48]. Serotonin is a multifunctional signaling molecule that mediates a number of cellular functions. It has been demonstrated that peripheral inflammation extends to the hypothalamus, where it affects serotoninergic metabolism [49]. These hypothalamic changes in the serotonin pathway provide evidence of a role for serotonin in inflammation-induced responses. In the present study, we observed a significant increase in serotonin levels in the plasma, and especially the brains, of mice with inflammation (Fig 6). In contrast, melatonin concentration was found to be reduced both in blood plasma and the brain, with a more marked decrease evident in the brain. Overall, our results highlight the fact that peripheral inflammation reaches the hypothalamus and pineal gland, affecting serotonin and melatonin metabolism. These observations agree with prior findings demonstrating a tendency for alterations in serotonin and melatonin levels upon inflammation [48, 49]. The partial restoration by thymulin of levels of both hormones in the tissues of mice with inflammation represents a major novel result in this context.

In conclusion, thymulin treatment of mice with inflammation alleviated fever, increased splenic cell number, and decreased cytokine production, Hsp72, Hsp90, and TLR4 expression, and NF-κB, p38 MAPK, JNK MAPK, and PKC-θ signaling pathway activity. In addition, thymulin restored levels of serotonin and melatonin in the brain and blood. PBCA nanoparticle-bound thymulin was more effective in modulating NF-κB, p38, and PKC-θ pathway activity, Hsp72 and Hsp90 production, hormone levels, and total splenic cell numbers. It should also be noted that according to the literature, the toxicity of the PBCA carrier is extremely low and only evident at very high doses, with its LD_50_ being 230 mg/kg [50]. Thus, the efficacy of thymulin as a co-therapy may be significantly improved by its being bound to PBCA nanoparticles, which prolong its residence time in plasma.

Currently, the mechanisms underlying the effects of thymulin are poorly understood. Almost no data exists concerning interactions between thymulin and receptors, although T-cell membranes are known to contain specific receptors that bind thymulin with high affinity [51]. Our data indicate that the effects of thymulin on immune cells may be exerted via the NF-κB signaling pathway, the activity of which is decreased by hormone administration [52]. This finding is in agreement with a prior report that p38 signaling pathway activity is diminished following thymulin administration [53]. Moreover, our own recent study showed that thymulin may be a damage-associated molecular pattern-like molecule released by various cell types in response to stress [54]. Thus, a better understanding of the mechanisms controlling signaling pathways may enable the identification of new therapeutic targets for the prevention of aberrant NF-κB, MAPK, and PKC-θ cascade activation in human SIRS and sepsis. Given the widespread belief among clinicians that it is preferable to prevent rather than treat sepsis, prior intervention with thymulin, a non-toxic, naturally occurring molecule, may be a promising approach to achieving this goal, thereby diminishing the risk of fatal consequences.

## Conclusion

This is the first study to demonstrate that thymulin, free or PBCA nanoparticle-bound, may dampen the proinflammatory response in chronic endotoxemia. In addition, our data indicate that PBCA nanoparticle-bound thymulin is more effective than free thymulin in p38; JNK, NF-kB; PKC; HSP72; HSP90; 5-HT; melatonin, but had similar effect in IL-6; TNF-α; IFN-γ; IL-10; IKK; TLR4, p53, and caspase-3. Since inflammation was induced for only a limited time (8 days) in the present work, we believe that the benefits of the nanoparticle form will be more pronounced in inflammation over a prolonged period.

## Supporting information

S1 Raw Data Raw data for Table 1. (ZIP)
